# A tale of two MADs: a case series

**DOI:** 10.1093/ehjcr/ytaf266

**Published:** 2025-05-29

**Authors:** Kamil Stankowski, Dario Donia, Diego Penela, Renato Bragato, Pier-Giorgio Masci, Gianluigi Condorelli, Marco Francone, Stefano Figliozzi

**Affiliations:** IRCCS Humanitas Research Hospital, Via Alessandro Manzoni, 56, Rozzano, Milano 20089, Italy; Department of Biomedical Sciences, Humanitas University, Via Rita Levi Montalcini, 4, Pieve Emanuele, Milano 20090, Italy; IRCCS Humanitas Research Hospital, Via Alessandro Manzoni, 56, Rozzano, Milano 20089, Italy; Department of Biomedical Sciences, Humanitas University, Via Rita Levi Montalcini, 4, Pieve Emanuele, Milano 20090, Italy; IRCCS Humanitas Research Hospital, Via Alessandro Manzoni, 56, Rozzano, Milano 20089, Italy; IRCCS Humanitas Research Hospital, Via Alessandro Manzoni, 56, Rozzano, Milano 20089, Italy; School of Biomedical Engineering and Imaging Sciences, Kings College London, St Thomas’ Hospital, London, UK; IRCCS Humanitas Research Hospital, Via Alessandro Manzoni, 56, Rozzano, Milano 20089, Italy; Department of Biomedical Sciences, Humanitas University, Via Rita Levi Montalcini, 4, Pieve Emanuele, Milano 20090, Italy; IRCCS Humanitas Research Hospital, Via Alessandro Manzoni, 56, Rozzano, Milano 20089, Italy; Department of Biomedical Sciences, Humanitas University, Via Rita Levi Montalcini, 4, Pieve Emanuele, Milano 20090, Italy; IRCCS Humanitas Research Hospital, Via Alessandro Manzoni, 56, Rozzano, Milano 20089, Italy; Department of Biomedical Sciences, Humanitas University, Via Rita Levi Montalcini, 4, Pieve Emanuele, Milano 20090, Italy

**Keywords:** Mitral valve prolapse, Mitral annular disjunction, Ventricular arrhythmias, Multimodality imaging, Coronary anomalies, Case report

## Abstract

**Background:**

Mitral annular disjunction (MAD), consisting in a systolic separation between the posterior atrial wall-leaflet junction and the basal left ventricular wall, is a disputed imaging entity. MAD was initially associated with sudden cardiac death and ventricular arrhythmias in patients with mitral valve prolapse, whereas in more recent studies, it has been presented as a normal variant of the mitral annulus.

**Case summary:**

In the present series of two cases, we show a case featuring a young woman with syncope that showed a microscopic MAD on echocardiography but, after a thorough multimodality assessment, was diagnosed with a coronary anomaly responsible for her presentation. In the second case, a young man presenting with aborted sudden cardiac death was found to have a macroscopic MAD in the context of mitral valve prolapse with numerous high-risk arrhythmic features.

**Discussion:**

The need for assessing the diverse significance of MAD in the clinical and imaging context of each patient is underscored. Assessment of MAD should be complemented by other imaging and clinical parameters: the circumferential and longitudinal extent of MAD, the presence of repolarization abnormalities or ventricular arrhythmias, bi-leaflet prolapse, systolic curling, the Pickelhaube sign, left heart remodelling, and the presence of myocardial fibrosis, among others.

Learning points

- Mitral annular disjunction is a systolic separation between the posterior atrial wall-leaflet junction and the basal left ventricular wall.
- Mitral annular disjunction was initially associated with ventricular arrhythmias and sudden cardiac death in patients with mitral valve prolapse, whereas recently it has been presented as a normal and highly prevalent variant of the mitral annulus.
- Mitral annular disjunction must be interpreted in the clinical and imaging scenario of the single patient, considering the presence of other high-risk features, while further evidence is awaited.

## Introduction

Mitral annular disjunction (MAD) is a systolic separation between the posterior atrial wall-leaflet junction and the basal left ventricular (LV) wall commonly found in patients with mitral valve prolapse (MVP). While some evidence supported an association between this imaging feature and the risk of malignant ventricular arrhythmias,^1,2^ contrasting data suggested MAD is a normal variant of the mitral valve apparatus.^3^ We present two paradigmatic cases exemplifying the ambivalent nature of MAD.

## Summary figure

ECG, electrocardiogram; ECV, extracellular volume

**Figure:**
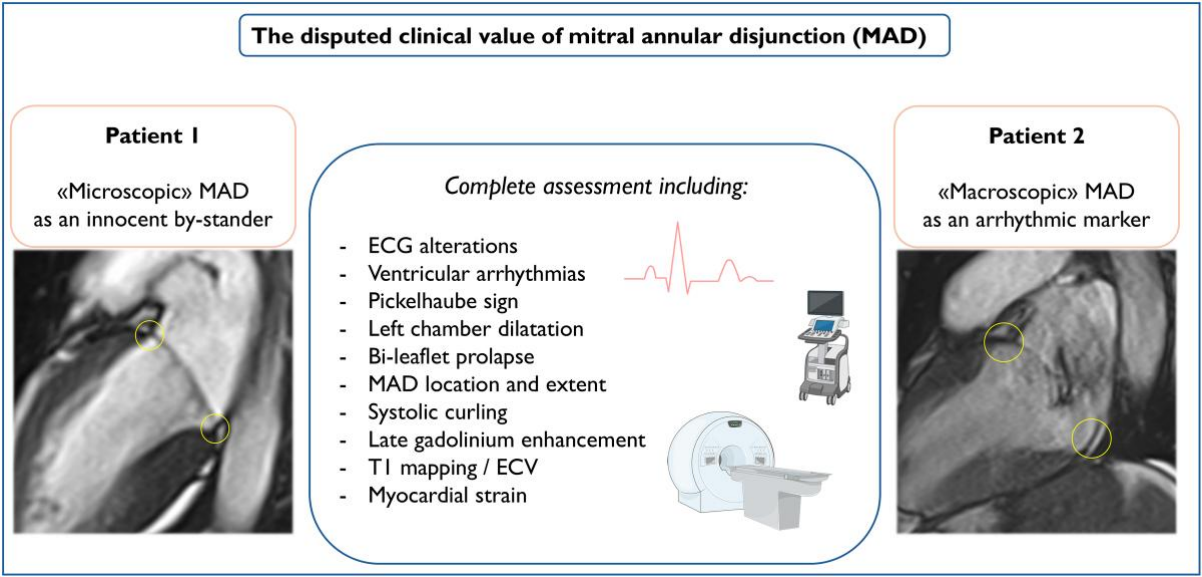


## Patient 1

**Figure 1:**
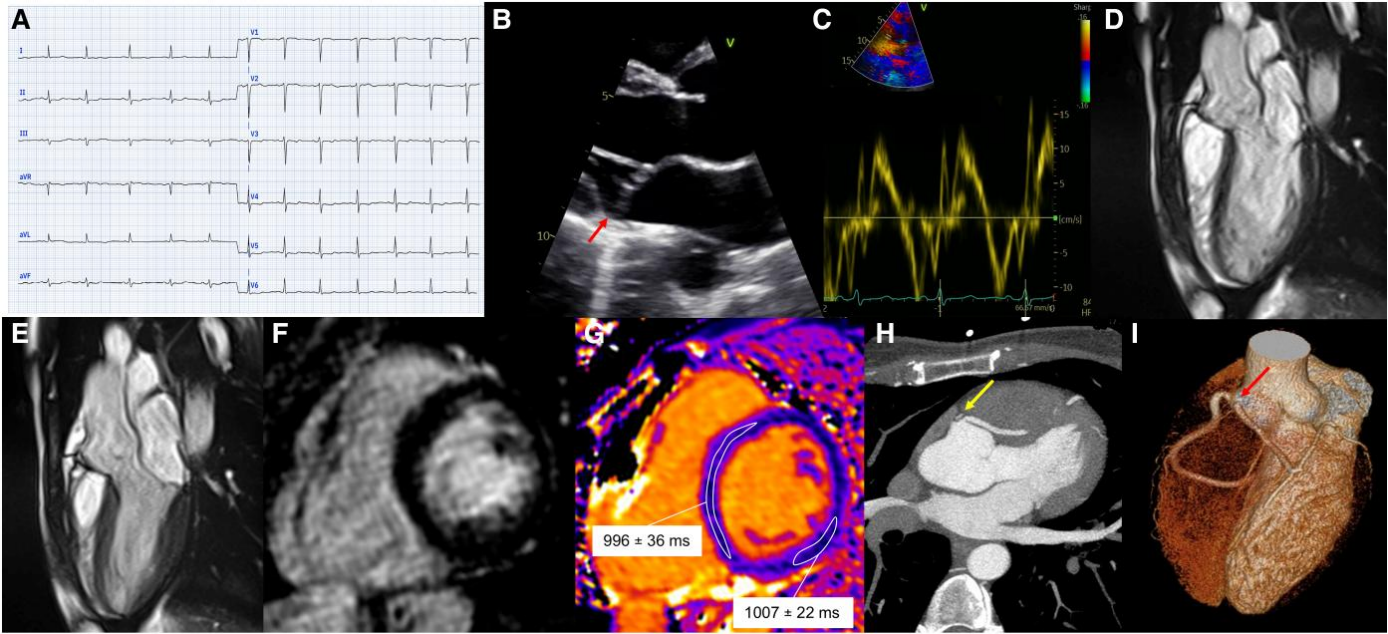
Electrocardiogram and imaging features of Patient 1. (*A*) Presenting electrocardiogram. (*B*) Echocardiography showing mitral annular disjunction of limited extent (*arrow*). (*C*) Tissue Doppler imaging without the Pickelhaube sign. (*D*, *E*) Cine-cardiac magnetic resonance end-diastolic and end-systolic three-chamber view frames. (*F*) Absence of late gadolinium enhancement. (*G*) T1 mapping map showing normal values in the septum and inferolateral basal walls. (*H*, *I*) Cardiac computed tomography showing abnormal origin of the left main from the right coronary sinus (*arrows*) with transseptal course.

Patient 1 was a 31-year-old woman admitted to our hospital because of exertional syncope preceded by sudden-onset palpitations. She had no known personal or familial history of cardiac disease. Blood pressure was 105/75 mmHg and heart rate 67 b.p.m. Baseline electrocardiogram (ECG) showed sinus rhythm and widespread ventricular repolarization abnormalities (*Figure 1A*). Laboratory tests were unremarkable, including high-sensitivity troponin I (5 ng/L; normal value < 18 ng/L). Echocardiography revealed normal biventricular function and normal chamber volumes. A single-leaflet non-classic MVP of limited entity was present with trace mitral regurgitation as well as MAD with a longitudinal extent of 3 mm in the parasternal long-axis view (*Figure 1B*), in the absence of Pickelhaube sign (*Figure 1C*). In the hypothesis of an ‘arrhythmic MVP’, the patient underwent cardiac magnetic resonance (CMR), which excluded systolic curling (see Supplementary material, *Video S1*) or tissue abnormalities: absence of focal fibrosis [late gadolinium enhancement (LGE)] and microscopic fibrosis (normal T1 mapping: septum 996 ± 36 ms, inferolateral wall 1007 ± 22 ms, reference < 1040 ms; *Figure 1D–G*). The MAD was 4 mm in the four-chamber view, 3 mm in the two-chamber view at the inferior wall and 1.5 mm at the anterior wall, and absent in the three-chamber view (*Figure 3A–C*). Cardiac computed tomography (CCT) showed an abnormal origin of the left coronary artery from the right sinus of Valsalva with a transseptal course with significant systolic compression of its lumen (*Figure 1H* and *I*), in absence of atherosclerotic plaques. In order to verify whether the anatomical abnormality we observed was correlated with the ischaemic symptoms of the patient, provocative ischaemia testing with instantaneous wave-free ratio (iFR) with dobutamine administration was performed. The test was positive at 30 μg/kg/min of dobutamine when if iFR was pathologically low at 0.88 (cut-off 0.89) in the left anterior descending artery (see Supplementary material online, *Table S1*).

Although an arrhythmic MVP phenotype was initially suspected, the absence of high-risk arrhythmic features on subsequent imaging exams made this aetiology much less likely. Despite the initial negative high-sensitivity troponin I, in light of the coronary anomaly found at CCT and subsequent positive ischaemia testing, the patient’s presenting symptoms and ECG with widespread repolarization abnormalities were considered of ischaemic origin and the patient underwent intraconal unroofing of the left coronary artery. The patient is doing well at 1-year follow-up.

## Patient 2

**Figure 2:**
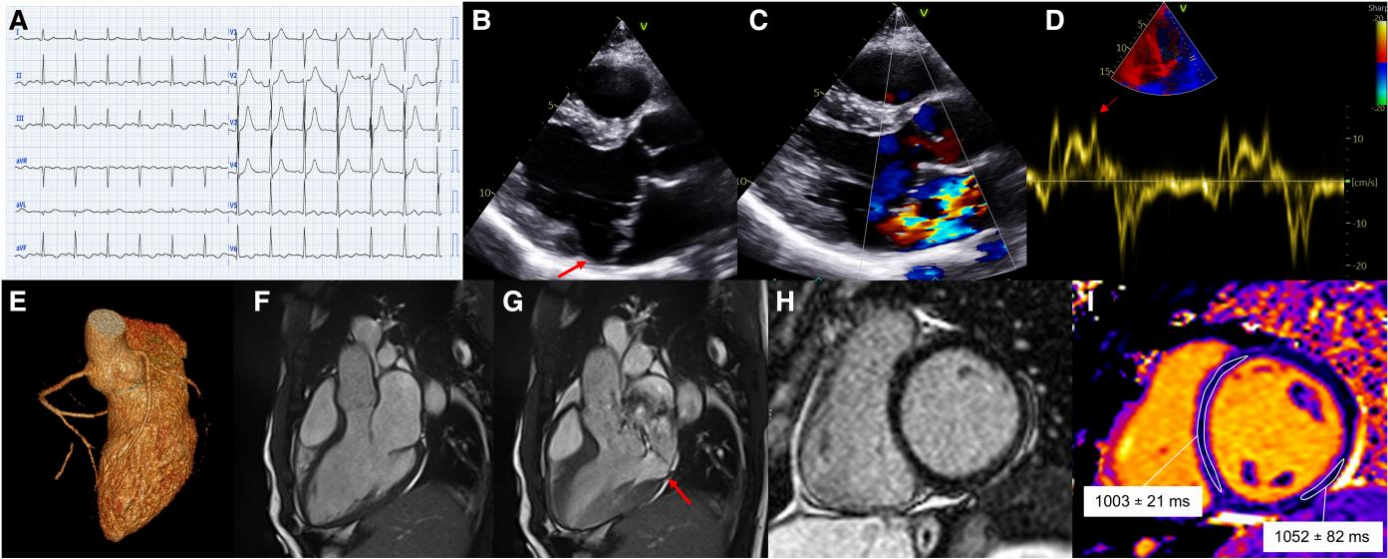
Electrocardiogram and imaging features of Patient 2. (*A*) Presenting electrocardiogram. (*B*) Echocardiography showing extensive mitral annular disjunction (arrow). (*C*) Colour Doppler showing mitral regurgitation. (*D*) Tissue Doppler imaging with positive Pickelhaube sign (arrow). (*E*) Cardiac computed tomography showing normal coronary arteries. (*F*, *G*) Cine-cardiac magnetic resonance end-diastolic and end-systolic three-chamber view frames showing extensive mitral annular disjunction (arrow). (*H*) Absence of late gadolinium enhancement. (*I*) T1 mapping map showing normal values in the basal septum, but elevated values in the basal inferolateral wall.

**Figure 3:**
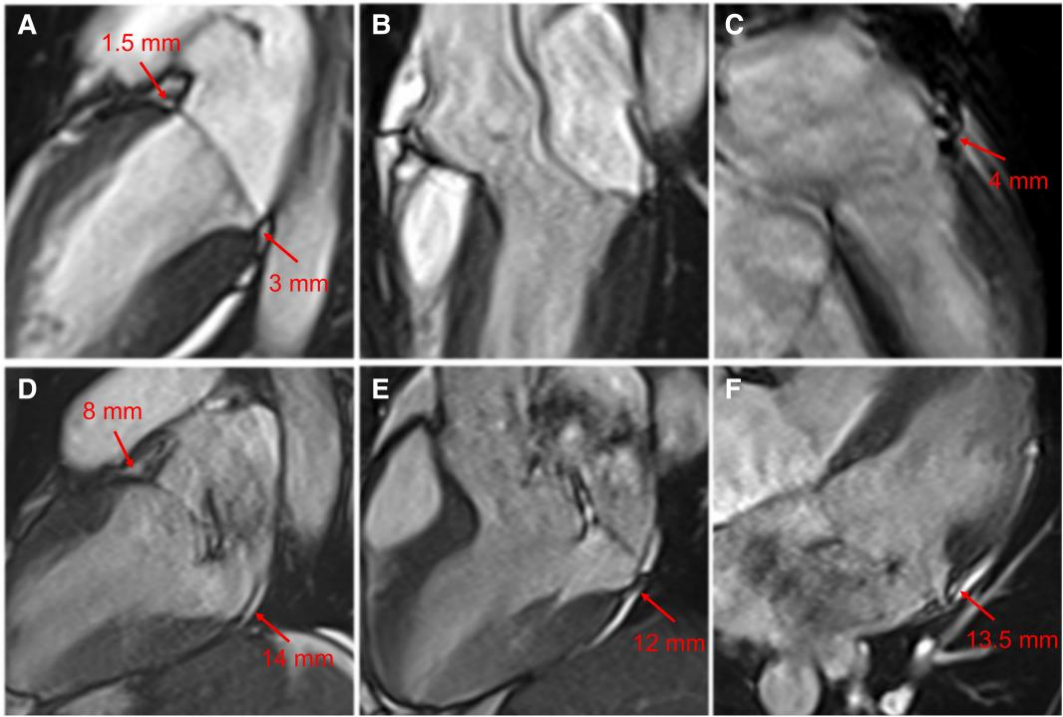
Close-up view of mitral annular disjunction in Patient 1 and Patient 2. Patient 1: cardiac magnetic resonance systolic cine frames of (*A*) two-chamber, (*B*) three-chamber, and (*C*) four-chamber view. A mitral annular disjunction of limited longitudinal extent (*arrows*) is evident in two-chamber and four-chamber views. Patient 2: cardiac magnetic resonance systolic cine frames of (*D*) two-chamber, (*E*) three-chamber, and (*F*) four-chamber view. A mitral annular disjunction of extensive longitudinal extent (*arrows*) is evident in all long-axis views.

Patient 2 was a 32-year-old man presenting with aborted sudden cardiac death due to ventricular fibrillation occurring during the recovery after a run. Return of spontaneous circulation was obtained after 5 DC shocks (no-flow 3 min, low-flow 14 min), and the patient remained haemodynamically stable with no inotropic support. At arrival in the emergency department, he was sedated, intubated, with blood pressure 124/78 mmHg, heart rate 88 b.p.m., oxygen saturation 100% while being ventilated with a fraction of inspired oxygen of 28%. The ECG showed sinus rhythm and T-wave inversion in the inferolateral leads (*Figure 2A*). Laboratory examinations revealed mild hypokalaemia (K^+^ 3.3 mmol/L) and metabolic acidosis (pH 7.31, bicarbonates 19.1 mmol/L) with hyperlactacidaemia (lactate 5.3 mmol/L), whereas high-sensitivity troponin I was 350 ng/L (normal value < 18 ng/L). Head and chest computed tomography were normal. He was transferred to the cardiac intensive care unit where he was successfully extubated the next day, without neurological sequalae. He had no known personal or familial history of cardiac disease. Echocardiography showed normal biventricular function (LV ejection fraction: 63%; tricuspid annular plane systolic excursion: 26 mm; fractional area change: 39%), moderate dilatation of the LV (end-diastolic volume index 98 mL/m^2^), and mild dilatation the left atrium (left atrium maximum volume index 40 mL/m^2^) with normal filling pressures. Tricuspid regurgitation was absent, and no other indirect signs of pulmonary hypertension were present. Bi-leaflet classic MVP in the context of Barlow disease with moderate mitral regurgitation was present, as well as a MAD measuring 10 mm in the parasternal long-axis view (*Figure 2B* and *C*). Systolic curling and Pickelhaube sign were noted (*Figure 2D*). Cardiac computed tomography showed normal origin and course of the coronary arteries (*Figure 2E*). Cardiac magnetic resonance confirmed Barlow disease with extended systolic MAD (13.5 mm in the four-chamber view, 14 mm in the two-chamber view at the inferior wall and 8 mm at the anterior wall, and 12 mm in the three-chamber view; *Figures 2F* and *G* and *3D–F*; Supplementary material, *Video S2*), without obvious LGE (*Figure 2H*), but with elevated native T1 values in the inferolateral basal wall (1052 ± 82 ms; basal septal T1 1003 ± 21 ms; normal values < 1040 ms; *Figure 2I*). Mitral regurgitation was quantified as moderate with a regurgitant fraction of 40% and regurgitant volume of 55 mL. On ECG monitoring, frequent ventricular ectopic beats with superior axis and right bundle branch block-like morphology and brief runs of polymorphic non-sustained ventricular tachycardia were observed. As his arrhythmic phenotype was considered prevalent in the clinical picture and the entity of mitral regurgitation was not yet severe, thereby itself justifying the occurrence of adverse events, the final diagnosis was ‘arrhythmic MVP’, and the patient underwent implantable cardioverter-defibrillator implantation. He is doing well from an arrhythmic point of view and is followed up to decide the optimal timing for mitral valve repair.

## Discussion

In this two-case series, we show the ambivalent nature of MAD. In the first case, a limited MAD was initially blamed for the arrhythmic presentation, despite further workup demonstrating its innocent, bystander role; in the second case, on the opposite, an aborted sudden cardiac death was associated with a greater MAD in the context of an arrhythmic MVP.

The clinical implications secondary to MAD are uncertain. Early evidence suggested an association between this condition and the occurrence of malignant ventricular arrhythmias at cross-sectional evaluation.^1,2^ However, it was not associated with adverse outcomes at clinical follow-up of patients with MVP^4^ and turned out to be a very common condition when assessed with advanced imaging modalities.^3,5,6^ Thus, it has also been suggested to represent a benign variant of the mitral annulus. Selection biases and methodological heterogeneity in MAD assessment stand behind conflicting evidence. In fact, in a recent study^5^ including 441 consecutive patients undergoing clinically indicated CMR, the prevalence of MAD in the study cohort dramatically reduced from 49% to 3% by increasing the diagnostic MAD cut-off from 1 to 6 mm. Interestingly, the MAD extent correlated with the MVP extent. Only MADs > 4 mm were associated with an increased burden of ventricular arrhythmias, whereas less extended MADs were not. Our cases are in keeping with these messages, softening the clinical importance of limited MADs. Moreover, our cases exemplify that MAD must be interpreted in the clinical and imaging scenario. Patient 1 showed a MAD isolated from other high-risk clinical and imaging features of SCD in the arrhythmic MVP phenotype that were present in Case 2. These features included advanced degeneration of the mitral valve apparatus, left heart dilation, suggestive ECG changes, and ventricular ectopic beats morphology. In such context, extended MADs are accompanied by an exaggerated motion (curling) of the basal LV inferolateral wall.^7^ The consequences of the secondary abnormal traction forces on the LV might include mechanical induction of ventricular ectopic beats and development of myocardial fibrosis, which may induce malignant ventricular arrhythmias.^1,7^ Although it resulted in being a risk marker for sudden death,^4^ LGE was absent in Patient 2. Patient’s age was an independent associate of LGE in a large cohort of patients with MVP.^4^ Thus, LGE might be missing in young patients at risk of malignant ventricular arrhythmias, such as Patient 2. T1-mapping abnormalities might be of added clinical value in these cases, given the higher sensitivity for detecting underlying interstitial fibrosis. Subjects with MVP often present longer T1-mapping values in the basal and mid inferolateral segments,^8^ and increased extracellular volume in the basal segments was associated with complex ventricular arrhythmias in a study,^9^ suggesting an adjunctive role for multiparametric mapping in patients without LGE. Notably, T1 mapping might intercept interstitial fibrosis in patients with MVP, which has been described in autopsy studies and can be missed by LGE imaging.^10^

In 2022, an expert consensus document was published to provide guidance regarding risk stratification and management of patients with arrhythmic MVP, while highlighting numerous gaps in evidence regarding this population.^11^

In conclusion, the present report of two cases highlights the *Janus Bifrons* nature of MAD, which must be carefully interpreted in light of its severity and clinical and imaging context. Arrhythmic risk stratification is challenging in patients with MVP. However, a constellation of markers is emerging as novel tools to aid in prognostication and must be systematically searched for in this population (*Summary figure*). The longitudinal extent of MAD is one of those markers, as the higher the extent, the greater its arrhythmogenic potentials. In line with this, an isolated MAD of a limited extent should be considered a benign finding.

## Supplementary Material

ytaf266_Supplementary_Data

## Data Availability

The data underlying this article will be shared on reasonable request to the corresponding author.

## Lead author biography

Dr. Stefano Figliozzi is a cardiovascular imaging specialist with advanced expertise in multimodality imaging, particularly in echocardiography and cardiovascular magnetic resonance (CMR). He holds Level 3 CMR certification from the European Association of Cardiovascular Imaging (EACVI) and has over six years of full-time experience in tertiary imaging centers. He currently serves as an Honorary Senior Research Fellow at King's College London and as a staff physician at IRCCS Humanitas Research Hospital in Italy. He is also a PhD candidate in Biomedical Sciences at Humanitas University.

Dr. Figliozzi has authored over 70 peer-reviewed publications, including more than 25 as first or senior author. One of his main research interests is arrhythmic mitral valve prolapse (MVP), with a focus on improving arrhythmic risk stratification. On this topic, he received the EuroCMR Young Investigator Award, authored a Section Expert Analysis for the American College of Cardiology (ACC.org), and delivered invited lectures at institutions such as Montefiore-Einstein. He has also served as faculty at major international congresses, including EACVI and SCMR.

He holds a second-level Master's degree in Advanced Biostatistics, he is a Nucleus Member of the Cardiac Magnetic Resonance Working Group of the Italian Society of Cardiology (2023–2025), a Fellow of the same society since 2024, and a member of the ESC Council on Cardio-Oncology.

## Supplementary material

**Consent:** The authors confirm that written consent for the submission and publication of this case report has been obtained from the patient following COPE guidance.

**Funding:** This work was partially supported by ‘Ricerca Corrente’ funding from Italian Ministry of Health to IRCCS Humanitas Research Hospital.
