# Correction to: A tale of two MADs: a case series

**DOI:** 10.1093/ehjcr/ytag001

**Published:** 2026-01-19

This is a correction to: Kamil Stankowski, Dario Donia, Diego Penela, Renato Bragato, Pier-Giorgio Masci, Gianluigi Condorelli, Marco Francone, Stefano Figliozzi, A tale of two MADs: a case series, *European Heart Journal - Case Reports*, Volume 9, Issue 7, July 2025, https://doi.org/10.1093/ehjcr/ytaf266

The third co-author's name is emended to read: “Diego Penela”.

