# Supplementary material for: A tale of two MADs: a case series
Source: Eur Heart J Case Rep. 2025 May 29;9(7):ytaf266. doi: 10.1093/ehjcr/ytaf266 (PMC12272160; doi:10.1093/ehjcr/ytaf266)
Supplement: ytaf266_Supplementary_Data [file ytaf266_supplementary_data.zip › Table S1.docx]

**SUPPLEMENTARY MATERIAL**

Table 1. Results of invasive provocative ischemia testing with instantaneous wave-free ratio (iFR) with dobutamine administration.

| Instantaneous wave-free ratio (iFR) | |
| --- | --- |
| Dobutamine dose | iFR (LAD) |
| Rest | 0.97 |
| 1 μg/kg/min | 0.96 |
| 5 μg/kg/min | 0.95 |
| 10 μg/kg/min | 0.93 |
| 15 μg/kg/min | 0.91 |
| 20 μg/kg/min | 0.88 |
| 30 μg/kg/min | 0.88 |

LAD: left anterior descending artery.

Video 1. Cine cardiac magnetic resonance 3-chamber view of patient 1.

Video 2. Cine cardiac magnetic resonance 3-chamber view of patient 2.
